# On the transmission of texts: Written cultures as complex systems

**DOI:** 10.1093/pnasnexus/pgag207

**Published:** 2026-07-07

**Authors:** Jean-Baptiste Camps, Julien Randon-Furling, Ulysse Godreau

**Affiliations:** École nationale des chartes, Université Paris, Sciences & Lettres, 65 rue de Richelieu, Paris 75002, France; Centre Borelli, ENS Paris-Saclay, 4 Av. des Sciences, Gif-sur-Yvette 91190, France; College of Computing, UM6P, Lot 660, Ben Guerir 43150, Morocco; École nationale des chartes, Université Paris, Sciences & Lettres, 65 rue de Richelieu, Paris 75002, France

**Keywords:** transmission of texts, complexity science, loss of cultural artifacts, stochastic models, evolutionary dynamics

## Abstract

Knowledge of past cultures relies heavily on surviving written material. Over the centuries, texts were copied, altered, and often lost, leaving scholars to reconstruct partial genealogies—*stemmata*—from shared innovations between surviving copies. Yet, a general understanding of the processes shaping textual transmission remains elusive. Within the broader topic of cultural evolution, text transmission provides a favorable context for integrating formal modeling with empirical evidence: explicitly identified items (texts), transmitted through deliberate manual replication, have left tangible artifacts (manuscripts)—traces of a dynamical, cultural, and historical process. Rethinking textual transmission through a complexity science approach, this study combines stochastic models and simulations, informed by historical scholarship, with empirical data from a corpus of circa 2,000 medieval manuscripts spanning four centuries. Our framework quantifies how variation in copying and destruction rates influences survival or extinction, and reproduces key stylized facts observed empirically in reconstructed stemmata, such as imbalance, a feature debated for over a century. Further, this approach provides broad trends estimates suggesting that up to 60%; of texts and more than 95%; of manuscripts may have been lost. Our findings highlight the role of drift in cultural transmission, while providing a formal basis to integrate drivers such as cultural selection and historical contingencies (eg the Black Death). It bridges philology and cultural evolution approaches, while providing a theoretical and empirical framework applicable to numerous other traditions—eg Classical literature, legal and scientific texts, religious canons—where replication and loss also shape what endures.

Significance statement Much of what we know about past cultures comes from the texts that happened to survive, yet a general understanding of the process of text transmission is still lacking. We present a general model that underscores the role of drift in cultural survival and creates a framework for exploring other driving forces. When confronted with empirical evidence, our model confirms that the transmission process of texts over the centuries is heavily marked by loss—with up to 60%; of texts and more than 95%; of manuscripts disappearing in our case. Our results expose the fragility of cultural heritage and offer new tools to understand how randomness, historical contingencies, and human choices have shaped the legacy we inherit today.

## Introduction

How did written works survive (1)? Why do we know the names of Gilgamesh, Ulysses, or King Arthur, and how many others have we forgotten? How much do we preserve of the written knowledge and cultures of the past? And how representative is what we know, compared to what existed? Such fundamental questions depend on the material process through which texts were produced, distributed, and ultimately transmitted or lost.

Before the advent of the printing press, written texts were circulated in manuscript form. In order to make the text available, the author would dictate it to a secretary, or write a draft on wax tablets, papyrus, parchment or, later, paper, and this original, authorial, manuscript would then have to be copied manually by a scribe in the form of a new manuscript, and then circulated. Copies could in turn be used to create more manuscripts, again by manual copying, perhaps by other scribes in other regions at a later date. During this process, successive innovations were introduced in the text, either mistakenly, or intentionally, to make the text more suited to its intended audience. These alterations in the written sequence forming the text could then be transmitted to its “descendants” by a given manuscript. Wear and tear, accidents, and fashions caused the destruction of some manuscripts, while others enjoyed the long life of library preservation. In the end, knowledge was lost, textual richness reduced, and some texts went extinct, while others gained traction and were eventually preserved for future generations.

**Figure 1:**
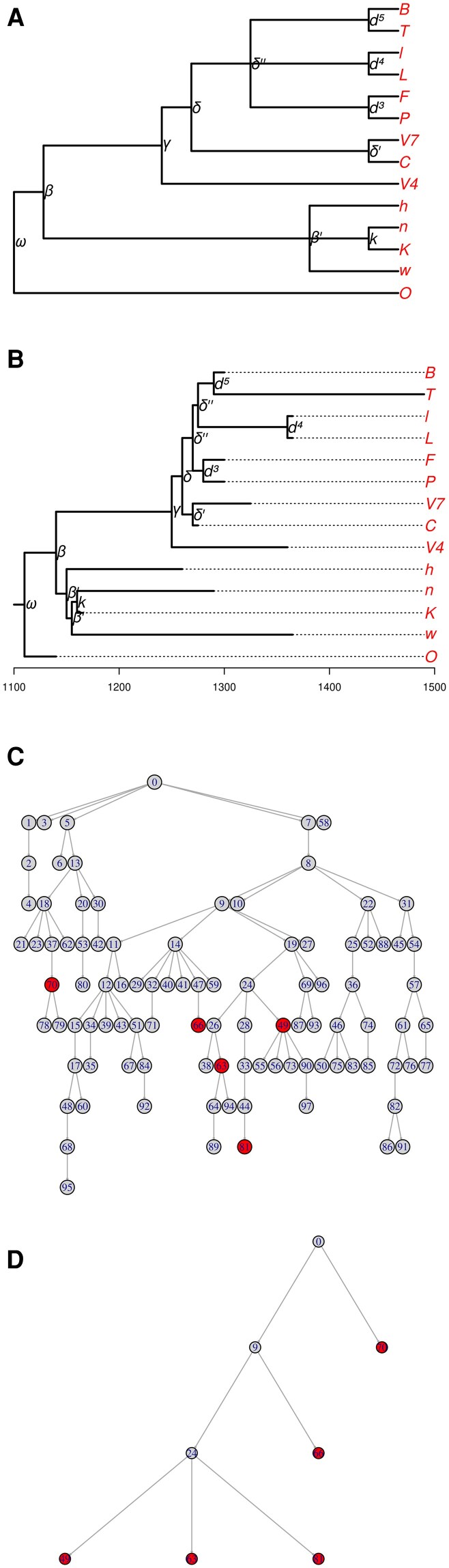
From the real tree to the *stemma codicum*. The stemma contains all witnesses, and as many hypothetical nodes as are needed to explain their relationships. A) Stemma of the *Song of Roland*, following Segre (5), roman letters (in red) stand for extant witnesses, and Greek letters for hypothetical shared ancestors; stemmata contain polytomies (nodes with out-degree >2) when a given ancestor has been copied more than twice, and more than two lineages of its descendants survive; B) in practice, a stemma codicum could be turned into a binary phylogram, by introducing time, and considering successive copies of a same manuscript as distinct events. C) Simulated complete tree of a textual tradition; the five dark red nodes depict surviving manuscripts (ie witnesses), and light gray nodes lost ones; D) reduced tree (*stemma codicum*) showing only witnesses, and their latest shared ancestors; this corresponds to the part of the original tree that could be inferred by philologists from surviving evidence.

Scholars have tried to make sense of the surviving documents, by ascertaining the network of relationships between them, by making assumptions about lost sources or by trying to reconstruct such lost sources. In the process of doing so, they have produced, since the 1820’s (2), evolutionary trees that philologists call *stemmata*. One of the staple methods introduced during the 19th century is the so-called “common errors” method (3, 4), whereby common innovations in the text appearing in surviving copies (called *witnesses*) of a work are exploited in order to infer genealogy-like relationships. From these innovations, a tree-type graph is obtained for the *tradition* (the set of manuscript witnesses that collectively preserve a given work), and this graph is called a *stemma codicum* (Fig. 1A). There is obviously a strong formal analogy between this approach and the way phylogenetic trees or cladograms are obtained in evolutionary biology or linguistics, based on shared characteristics between observed or inferred species. More recently, computational methods were directly imported from these fields to construct stemmata of textual traditions (6, 7), either through deterministic methods such as maximum parsimony (8), or Bayesian inference procedures (9–11). In this latter case, the reconstitution of the tradition’s history relies on the definition of prior stochastic models of text reproduction, the relevance of which have not been fully assessed. Current limitations include restrictions to binary trees (Fig. 1B) or continuous evolution of variants along branches, poorly approximating the intrinsically discrete and polytomic nature of manuscript transmission.

Beginning with the work of scholars such as Boyd and Richerson (12), a field studying cultural evolution has progressively emerged, building a methodology that combines formal modeling of culture with the analysis of empirical evidence (13). In the specific case of textual data, investigations have engaged with philogeny and ancestral state reconstructions of folktales and oral traditions (14–16), or the large scale evolution of thematic features in literature (17, 18), often with a functionalist perspective, or a priori cognitive or psychological hypotheses. In comparison to these cases, the transmission of texts in manuscript form present several interests, that facilitate its modeling as a process of successive replications, and its confrontation to empirical data. Indeed, unlike in the case of the transmission of nonmaterial or abstract entities, manuscript transmission involves empirically defined objects (the texts), whose transmission through replication is not merely a metaphorical process but a literal one: the deliberate, manual replication of material artifacts by copyists. This materiality sets it apart, as we possess physical objects—the manuscripts themselves—that document at least some stages of the transmission process. In addition to textual innovations, more external evidence (eg mentions of their source by scribes, contracts, medieval library inventories or university regulations) abundantly attest of this process.

The modelization of manuscript tradition thus appears as an ideal case study to precisely evaluate the role of drift and sheer materiality in cultural diffusion processes (19).

In this regard, an interesting observation about the structure of stemmata was made by French philologist Joseph Bédier in 1928 (20): most trees reconstructed by philologists exhibit root bifurcation, ie a root with out-degree 2. Bédier was quick to attribute this feature to a bias in the comparative approach, spurring a century long methodological schism in textual scholarship (21, 22). Previous modeling research has often approached the question of bifidity by using purely combinatorial approaches or by applying decimation to static trees (23–28). Yet they were generally focused specifically on the property of bifidity alone, and have left aside the dynamic nature of the transmission of texts. Besides, large asymmetry (or imbalance) between the size of branches (Fig. 1A and B), a phenomenon pervasive in evolutionary biology (29), is also observed in many stemmata. These topological properties need not stem from biases in the reconstruction method itself, nor from historical externalities or selection pressure but could instead emerge from the transmission process’s own dynamics.

Another question of interest is the estimation of the amount of lost literature. A variety of methods have been used to estimate the loss of documents, drawing from the items surveyed in medieval catalogs (30), or using methods inspired by paleodemography (1) or ecology. In particular, recent work by Kestemont et al. (31, 32) used unseen species methods to estimate the “unseen” (ie lost) part of medieval chivalric literatures, treating works as species, individuals as documents, and contemporary libraries as observation sites. However, the unseen species method assimilates the survival of manuscripts to an unbiased sampling of all manuscripts that have existed and treats manuscripts that were produced at very different dates as having the same probability of being sampled. Yet, through the sheer passage of time, older manuscripts (and older texts) are less likely to have survived than more recent ones. Moreover, the existence of recent copies is dependent on the availability, at the time of their creation, of surviving manuscripts of a given text. Besides, the question of loss and survival is not a merely quantitative one but also pertains to the internal history of textual traditions, as loss of documents can prune away entire branches (Fig. 1C and D) of their full transmission history. In this sense, the problem of transmission, survival and extinction of texts is one of loss of evolutionary history, as is encountered also in evolutionary biology (33).

We present here a stochastic modeling and simulation framework that accommodates dynamical phenomena while allowing us to investigate the aforementioned properties and to estimate observed as well as unobserved variables. In so doing, we take what one may call a complexity science approach to philology.

**Figure 2:**
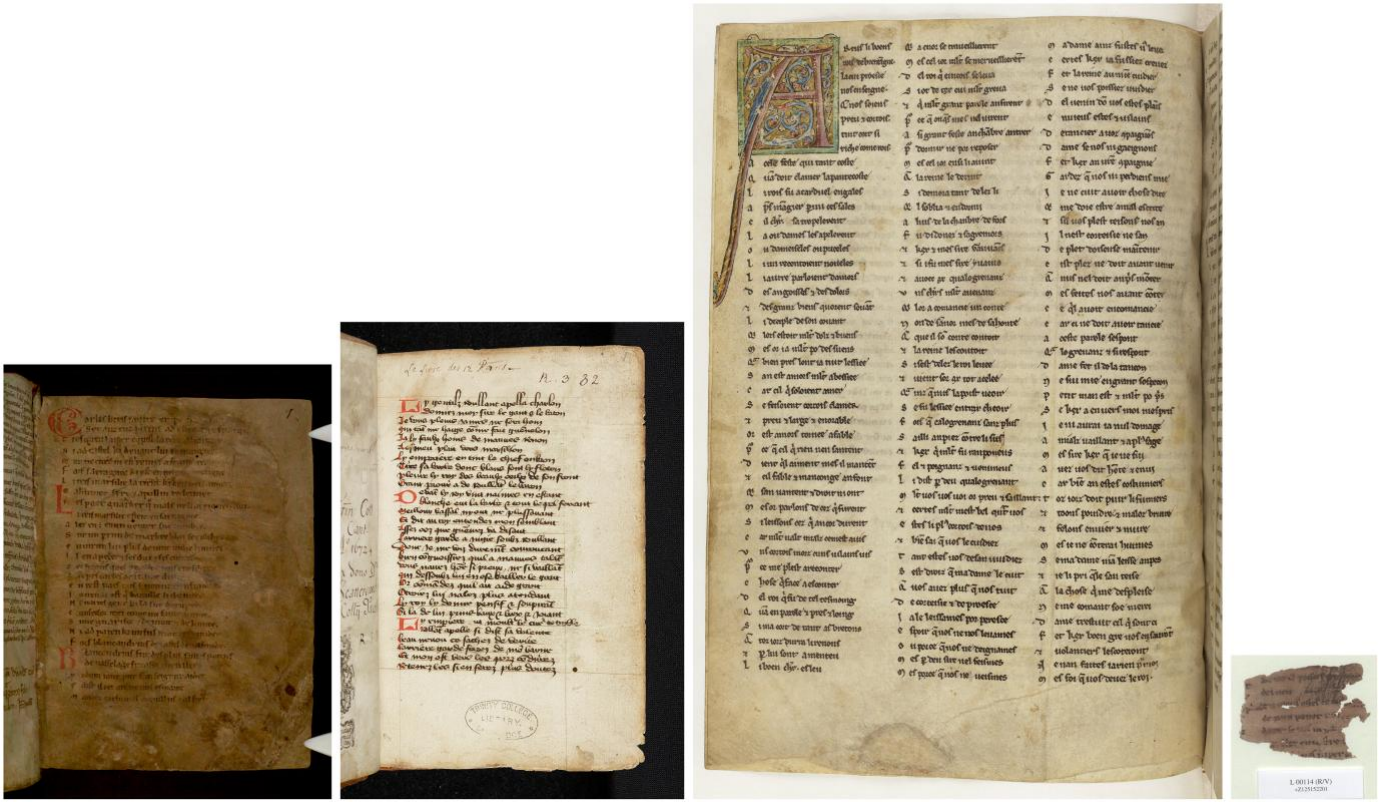
Old French chivalric narratives around Europe (years 1100–1500); A) Beginning of the *Chanson de Roland* (assonanced version, composed around 1090, maybe in Normandy), here in a manuscript copied in England (Oxford?) around 1130 (Oxford, Bodleian Library, Digby 23, Part 2, fol. 1r, h=175 mm); B) *Roland* rhymed version, copied in Western France, after 1431 (Cambridge, Trinity College Library, R. 3. 32, fol. 1r, h=192 mm); C) Beginning of Chrétien de Troyes’ *Yvain ou le Chevalier au lion* (originally composed around 1177 at the court of Marie de Champagne), in a parchment manuscript copied by the scribe Guiot in Provins (Champagne), around 1235 (Paris, BnF, fr. 794, fol. 79v, h=320 mm); D) fragment on paper of the same text, likely copied in Egypt in the middle of the 13th century (Wien, Österreichische Nationalbibliothek, L 00114 Pap, h=58 mm). Scale: 1/3.

We take a well-known case study, that has been subject to renewed attention lately (32). It concerns the beginnings of modern European literature, through the emblematic case of chivalric narratives, the heroic tales of Charlemagne, Arthur or Alexander and their knights, that emerged in French during the 12th century and then spread across Europe. Advantages of this case study include its geographical and chronological reach, as well as its central cultural importance. Indeed, these narratives were widely circulated in an area ranging from England to the Near-East (Fig. 2), and subsequently translated in many European languages as diverse as Norse, Irish, Castilian, or Middle High German, in a period extending for more than four centuries. Moreover, a large part of them has been well studied, from the 19th century onward, and is still accessible in public institutions, facilitating the collection of exhaustive information on them such as list of manuscripts, dates, and stemmata (Methods).

Although the generality of the model we consider makes it applicable in principle to various literary corpora and manuscript cultures, there are several advantages in taking a geographically, chronologically and thematically well delimited corpus of texts. First, it ensures a certain level of homogeneity in the material conditions of production and circulation of manuscripts. Moreover, the fact that our case study encompasses only a limited proportion of all manuscripts produced at any time makes the amount of produced manuscripts less dependent on the total production capacity existing at a given time, and more on the availability of sources for reproduction, as it is assumed in our model. Indeed the overall production of books, regardless of the nature of the texts, is expected to be limited by the size of the book market (in terms of production and consumption capacity) in a given area at a given time.

## Results

We ran agent-based simulations of birth-and-death manuscript traditions, as described in Methods, with a total time frame of 500 pseudo-years, of which $T_{\mathrm{act}}=250$ active, and $T_{\mathrm{inact}}=250$ inactive (in which manuscripts can be destroyed, but no longer copied), corresponding roughly, for the first part, to the time from the beginning of the 13th century, seeing the rise of vernacular fiction, to the introduction of the printing press during the Renaissance, and, for the second part, to the time between the Renaissance and the beginning of modern cultural heritage conservation efforts.

To assess the robustness of our model—and of the estimates it provides—despite its generality and minimalism in terms of historical motivations, we tested the model for tree scenarii reflecting historical dynamics, through a variation over time of the dynamical parameters during the active phase, emulating either intrinsic or extrinsic factors.

- **Constant rate model**: In the first scenario, the birth and death rates keep constant values over the active phase of the process.
- **Decaying popularity of texts**: In a second variant, we let the birth rate *λ* decrease linearly to zero over the active phase as $\lambda_{t}=2\lambda_{0}(1-t/T_{\mathrm{act}})$. Texts are supposed to be more fitted for the audience existing at the time of their creation, and this fitness then progressively declines.
- **Crisis-induced decimation**: In the third setting, we impose a sudden decimation of a half of living manuscripts in the tradition at $t=T_{\mathrm{act}}/2$. This variant attempts at modeling the effects of a large crisis event affecting the production and survival of manuscripts over a short period of time.

The historical reality is likely a combination of these dynamics, but we prefer here to study their effect independently rather than imposing ad hoc time-variations of parameters.

### Parameter values

**Figure 3:**
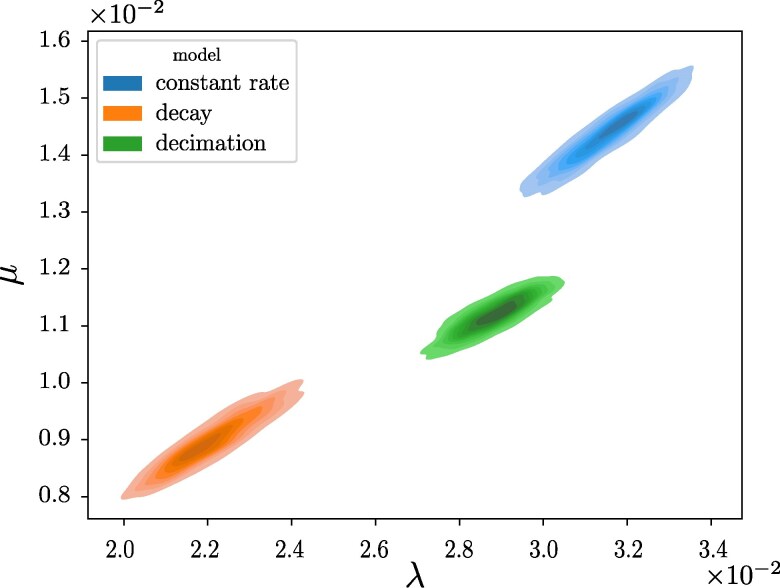
Posterior probability density of parameters (λ,μ) (kernel density plot for 1,000 samples) as obtained by simulation-based inference for the birth–death model in the tree settings considered: i) constant rate birth–death model (blue), ii) linear decay of *λ* (orange) iii) punctual decimation at half active phase duration (green).

We then perform simulation-based inference of parameter values for the three scenarii (Fig. 3). Results show that the posterior probability density of the parameters is clearly distinct for the three cases, yet their values are in the same order of magnitude and the ratio $\frac{\lambda }{\mu }$ they present is similar, between 2.3 for the constant-rate model to 2.6 for the decimation scenario (Table 1).

**Table 1.** Inference of nonobservable values from the parameters estimated through SBI for the three models.

	Constant-r.	Decay	Decimation
$\lambda \times 10^{-2}$	3.2	2.2	2.9
$\mu \times 10^{-2}$	1.4	0.9	1.1
λ/μ	2.3	2.4	2.6
Survival r. - works (%)	42	66	48
Survival r. - witns (%)	1.4	4.4	3.3
Half-life - witns (years)	22	33	27
Original-archetype dist.	2.3	1.3	2.0
Chao1 - works (%)	87	90	89

This table gives the value of *λ* and *μ* estimated through simulation-based inference for the three models, as well as values inferred, using the three models with these parameter values, for a set of statistics that are not observable in historical data: the survival rates of works and witnesses, the half-life of witnesses (years), as well as the median distance between the original and the archetype (latest common ancestor) of a tradition. Finally, we give the value of the Chao1 estimator, for the upper bound of the survival of works, to be compared with the true survival rate.

These results mean that on average, a manuscript would have been copied between two and three times during its average lifespan. The half-life of manuscripts would be log(2)/μ years, approximately between 20 and 35 years for our inferred parameters. The half-life of all texts, on the other hand, would be close to 500 years (in accordance with survival rates), but only around 40 years for the texts that ultimately did not survive.

Posterior predictive checks (Table S5) also show that the three scenarii yield traditions that have similar properties, in terms of population size, lifespan and topology, and that are comparable to traditions empirically observed.

Globally, the results obtained through simulation-based inference show the robustness of the model to variations accounting for different historical dynamics, and the relative stability of the results obtained for the aspects of textual transmission on which we are investigating.

### Loss estimates

In terms of losses, our three scenarii yield results comprised between 1.4 and 4.4% for the survival of witnesses, and between 42 and 66% for the survival of works (Table 1). These estimates are mostly compatible with the upper-bound of survival provided by Kestemont et al. (32) using the Chao1 species richness estimator (34) for the same corpus (resp. 5.4 and 53.5% as upper bound of survival). It is to be noted that both loss estimates for manuscripts are in the range or close to the global loss rate of nonillustrated manuscripts of 93–97% estimated by historians based on external evidence (see Supplementary material). Interestingly, if the Chao1 estimator had been applied to our simulation results, it would have overestimated survival by a factor of up to 2 (Table 1), showing that there are contexts in which the Chao1 estimator gives an upper bound of survival that can be substantially different from actual survival rates and can lead to underestimating losses. The decay model shows a remarkably higher survival rate of works than the two other variants. This hints at the importance of the first few years in the ultimate survival of a work. During this period, shortly after its creation, when very few copies are in circulation, a work can easily completely disappear. If the production is higher during this initial phase, for instance if the work quickly encounters success or if its author succeeds in having an important number of copies produced quickly, this might help the work leave this early “danger zone” and increase its odds of surviving the next centuries.

The fact that our estimates of loss are close to those obtained with a Chao1 estimator on historical data (32), while the application of the same methods to the simulation results would underestimate the loss significantly, can be explained by differences in terms of distribution of witnesses per work. Indeed, our model creates data that follows an exponential distribution, while they have, in real data, a power-law type behavior. A large fraction of the total population has a vanishingly small probability of being sampled at any rate in the case of an exponential distribution of witnesses per text, which causes the Chao1 estimator to largely underestimate the size of the underlying population.

### Tree topology, bifidity, and imbalance

Beyond the mere probability of survival or extinction, our results also show that the trees that philologists can draw from surviving witnesses often represent a localized subset of the original tree: the loss of complete branches is apparent in the average distance of ∼2 generations between the original and the latest common ancestor of the surviving tradition, the archetype for most models (Table 1). This means that, in most cases, the original state of the text is not accessible through the preserved tradition, that does not extend beyond a later archetype. It implies also a loss of the text’s evolutionary history, through the disappearance of collateral branches that are entirely missing from the preserved tradition. Still, the decay scenario, that exhibits a shorter original-archetype distance, hints that this dynamic is sensitive to time variation. Maybe counter-intuitively, texts with a sustained and stable popularity could be recoverable to us only in a more evolved and innovated form, more remote from the original work of the author, than texts whose initial popularity faded quickly and whose copies were produced in greater numbers early on. This echoes the situation of texts, such as the *Song of Roland*, whose oldest versions are probably completely lost, and that survive mostly in later reflections or rewritings. Here too, we can hypothesize that, for medieval authors, supervising the initial production of copies of their work could have been a relatively successful way of improving its availability in a less altered or innovated form.

Finally, when properties related to imbalance are examined, results show that the constant-rate and decimation models produce trees with levels of root-bifurcation and internal polytomies that near those observed in philological reconstructions. Yet they still produce trees that are substantially less imbalanced (Table 2).

**Table 2.** Comparison of various topological properties for real stemmata and simulated ones for inferred values of parameters.

Property	Bifidity	$i_{3}$	P(degree≥3)
Empirical	**77%** $\begin{array}{c} 86\%\\ 65\% \end{array}$	**0.93** $\begin{array}{c} 0.96\\ 0.89 \end{array}$	**0.36** $\begin{array}{c} 0.38\\ 0.32 \end{array}$
Constant-rate	65%	0.83	0.33
Decay	57%	0.76	0.31
Decimation	67%	0.84	0.32

Upper and lower bounds for our estimations on real world data are computed from the 10% confidence interval of the bootstrap distribution over our corpus of 117 stemmata, with 100 resamplings. *Bifidity* refers to the proportion of trees with root degree two. The degree probability P(degree≥k) is restricted to internal (nonleaf) nodes. As for the definition of $i_{3}$ imbalance, see Methods.

## Discussion

### Solving Bédier’s paradox and providing loss estimates

We have shown how relatively simple stochastic processes, that can be computer simulated, can reproduce the properties observed in historical data, specifically for the textual traditions of medieval French stories about Charlemagne, King Arthur, and the fall of Troy. Our results are consistent with those obtained through different methodologies, such as those recently published by Kestemont et al. (32). Our work incorporates previous work by Weizmann and Cisne (1, 35) and provides a much more general framework in which it is possible to refine and tune details from a simple birth-and-death process to a heterogeneous agent-based model calibrated via machine learning methods. This approach allows us to account for population dynamics in time, for loss and production estimates, as well as for certain structural properties of the trees of texts (*stemmata*). In particular we solve Bédier’s century-old paradox (20), that has been, to this day, at the core of a lasting schism in philological studies.

Indeed, our model shows that a simple, parsimonious stochastic process, without any of the supposed philologists’ methodological bias that Bédier assumed, produces a ratio of tree root bifurcation around 57–67% for the parameter values inferred using historical data (Table 2). This ratio shows that bifid stemmata are to be expected as the most frequent topology of tree for historical traditions, a fact congruent with existing philological reconstructions.

While this ratio is slightly lower in simulations than in historical data, our framework is easily extensible to include additional refinements, such as heterogeneity in the copy and destruction rates of manuscripts (based on chronological, spatial, or typological determinants), meso-scale destruction corresponding to historical events, or speciation processes (textual derivation and intertextuality), that could lead to higher ratios of root bifurcation.

A second important result obtained through our general framework is a set of estimates for the loss of texts and manuscripts across centuries—these are the first estimates based on computer simulations of artificial manuscript populations. Consistent with the upper range of loss estimates provided by external historical evidence (see Supplementary material), our results bring new insights into the vast continent of lost cultures and establish a link between the immaterial loss of cultural diversity (both qualitatively and quantitatively) and the disappearance of entire branches from the trees of texts, as well as a relatively high ratio (up to 58%) of fully missing trees. This makes evident how partial and fragmented our perception of past cultures may be.

Further, our results show the importance of taking into account diachrony and the evolutionary nature of text transmission. In addition to a better understanding of the process of text transmission itself, these results tend to indicate how a purely synchronous approach, as the one with ecodiversity methods, could lead to estimates relatively far from actual losses in time, and would not allow us to fully grasp the way in which loss is distributed. This is an important aspect, as the loss of entire branches (let alone entire trees) has a very different impact in terms of loss of textual history than the mere loss of individuals in surviving lineages. Moreover, the substantive differences observed in the results of the decay scenario, in terms of depth and work survival, shows that at least some variation in time of the copy rate can have important impacts.

### Beyond drift: selection, speciation, and extrinsic factors

Our results clearly show how much of the transmission, survival, and extinction of texts can be explained by drift, and how some of the salient properties of textual traditions, in terms of chronology, size of traditions or topological features of stemmata, may in large part be explained by the role of chance. Yet, they also point to the existence of other potential factors that need to be accounted for, such as the interrelation between texts and the possible role of selection and extrinsic factors.

**Figure 4:**
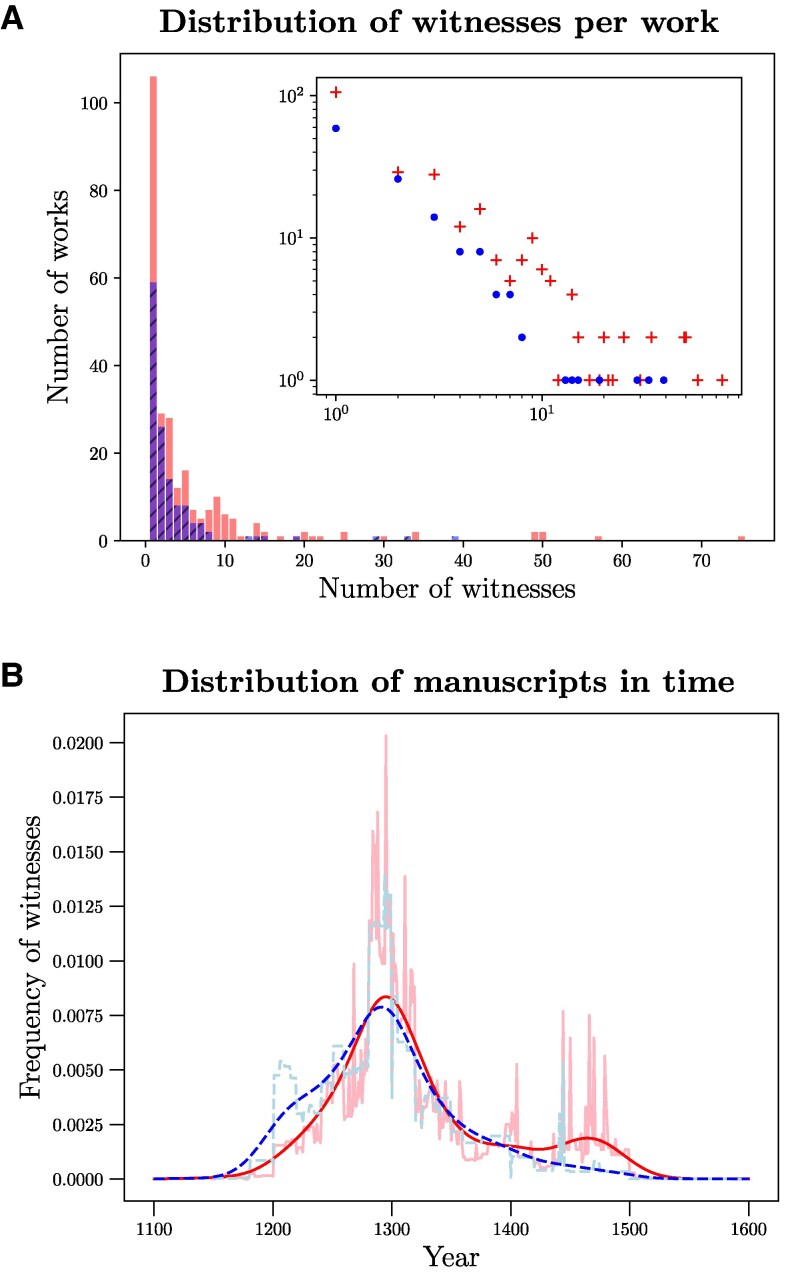
Time and abundance distributions of manuscript witnesses. A) The distribution of the number of witnesses per work shows a Pareto-like, heavy-tail behavior which does not correspond to the exponential distribution produced by our first level model (hashed blue bars and dots for fragments; full red bars and crosses for fully surviving witnesses). B) Distribution of witnesses production date for historical data (blue dashed lines for fragments).

Indeed, considering texts as independent entities does not fully reflect the way texts were created, very often by a process of derivation from preexisting texts, by translating, adapting, or rewriting existing material. This might be apparent in the way our model gives an exponential distribution for the number of witnesses per work, while real data appear to follow a Pareto-like heavy-tail behavior (Fig. 4A). The type of distribution observed is an intrinsic property of the qualitative dynamics of the model, and this discrepancy suggest that some important aspects of the dynamics of actual text diffusion are not captured—this does not come as a surprise, since the complex systems methodology voluntarily starts from the most basic model possible, before climbing one step at a time up the ladder toward more sophisticated models, in order to see which ingredients suffice to produce each observed property. In our case, accounting for the apparition of new texts from existing ones, through a process of speciation, might be enough to capture the heavy-tail distribution of the number of witnesses in historical data. A famous and widely used model including speciation, the Yule process (36), is known to produce power-law type distributions, and was originally aimed at understanding the power-law distribution of species per genus.

Additionally, our results point to the importance of extrinsic factors and perhaps also of selection. In our simulations, the distribution in time of witnesses grows exponentially, but historical data exhibit other kinds of variation (Fig. 4B), with ups and downs reflecting literary trends or external events, such as the Black Plague of 1346–1353, the Hundred Years’ War or more generally the economic and demographic crisis of the late Middle Ages (37). Models including selectivity, or accounting for extrinsic factors and nonrandom drivers of extinction—similarly to climate change in biology (33)—could help account for these discrepancies. The two alternative scenarii presented above represent a very first step in this direction.

In fact, the decay variant, tailored to account for a copy rate highest at the time of the creation of a work, and declining afterwards, already hints at the importance of the initial phase in the ultimate survival of a work, and its availability in a more stable and original (ie less innovated) form. As mentioned above, these properties can echo qualitative historical observations. In particular, some medieval authors are known to have supervised—or even directly taken part in—workshops dedicated to the copy of their own works, like Hildegard von Bingen or Christine de Pizan (38). This strategy, to organize themselves the initial production of copies of their works might have been sensible and successful from an evolutionary perspective, when the results of the decay scenario are examined (increased work survival rate, reduced depth of the stemma).

Moreover, if our simulations solve Bédier’s paradox on tree root-bifurcation, they still produce trees that are relatively less imbalanced than actual stemmata (Table 2). This is a problem that is also observed in evolutionary biology (29, 39, 40), and constitutes a further indication that drift alone cannot fully explain the process of text transmission, or hints at biases such as variation in copy and destruction rates between lineages or geographical regions where these lineages appear.

Another possible future direction is to account for evidence not strictly falling into the categories of survival or loss: in particular, a large number of fragments (a few leaves of otherwise lost manuscripts) have been preserved across time. They may not be considered cases of survival, yet they give empirical proof of the existence of lost manuscripts, in a way similar to fossils in paleodemography^a^. Yet, existing evidence still points to the fact that fragments behave similarly as surviving witnesses in terms of distribution, and do not radically challenge results obtained only on surviving witnesses, while being slightly older in terms of date, reflecting the cumulative damage in time affecting manuscripts (Fig. 4A and B).

Our results also confirms the relevance of polytomies in the evolutionary histories of texts, a phenomenon overlooked by most models used in phylogenetic inference. Indeed, more than 30% of internal nodes of empirical stemmata have out-degree larger than 3, and simulations suggest that in most cases, these corresponds to actual, *hard polytomies*, and not unresolved sequences of bifurcations (Table 2).

Finally, the generality of our framework and the models it can accommodate makes it applicable not only to medieval texts, but to any type of written transmission. Further investigations should include the broadest possible range of cases, starting with Western Medieval and Antique texts, but preferably also encompassing cultural productions from very different time periods and geographical areas.

## Methods

Due to the complex nature of the objects considered—namely manuscript genealogies over large periods of time—we base our approach on an agent-based stochastic model reproducing the elementary mechanisms of manuscript text transmission with a minimal set of a priori assumptions on the nature of these processes. The use of agent-based models (ABM) and computer simulations thereof have proven to be a remarkably successful investigation tool for various questions in cultural diffusion, with applications ranging from historical linguistics (41, 42) to formal evolution of prehistoric artifacts (43).

Weitzman articles (35, 44) have pioneered the use of stochastic agent-based models for manuscript transmission, but for a limited number of scenarii constraining parameters to predefined values motivated by historical extrinsic factors. We shall in fact elaborate on Weitzman’s work, using the same elementary dynamics, although in a more exploratory approach, by trying to fit the parameters of the model against observable features, rather than surmising them based on sparse historical data.

Our approach combines stochastic agent-based models, with machine-learning techniques. We use stochastic processes that are analytically tractable in their simplest form, and can always be computer simulated, in order to generate tens of thousands of artificial manuscript populations governed by the copy rate and the loss rate of manuscripts. Stemmata are then constructed for these artificial populations via an algorithm that emulates a philologist’s work on surviving witnesses. Heatmaps are obtained for various observables (eg the bifidity rate among stemmata) across ranges of values for the copy and loss rates. They are then compared to historical data, gathered for medieval traditions on observable features, using simulation-based inference (45). This ultimately allows us to make inferences for features that are not historically observable (such as loss rates).

### Approach

The specific process of text transmission—that is, the copy and circulation of manuscripts—varied through time and space, from on-demand production of a single copy by an individual (amateur or professional) copyist to forms of serial production in dedicated workshops. Instead of trying to account for all details, complexity science follows statistical physics in opting for a parsimonious type of modeling: the idea being that some, if not all, of the fundamental properties of a system will most likely not depend on a host of details but rather on only a couple of variables. Hence, it is relevant to start with the simplest possible model and examine what properties it already exhibits. Here we abstract all details away except the fact that manuscripts underwent copy (birth) and/or destruction (death).

From the original (authorial) manuscript, each new manuscript is produced by copying a single available manuscript. Of course, in historical cases, multiple ancestries and horizontal transmission (called contamination, in philology) are suspected to have existed. Yet, they appear to have remained sufficiently rare so that the transmission history of texts can in most cases be represented as a tree (in our dataset, if 28% of the stemmata present at least one case of horizontal transmission, only 2% of the nodes in total have more than one parent).

This leads to a stochastic process called a *birth and death* (BD) process, a special case of a Markov chain (46). In a BD process, individuals (here manuscripts) appear (“are born”) and are lost (“die”) at certain rates, corresponding to the *copy* or *birth* rate (*λ*) and to the *loss* or *death* rate (*μ*) of manuscripts. Thus, a population initially consisting of a single original manuscript evolves through time according to the following rule: at every time step t→t+Δt, each member of the population that is present at time *t* has probability λΔt to engender a new individual (a birth event) and probability μΔt to disappear (a death event), where rates $\lambda ,\mu \in R_{+}$ are nonnegative real numbers.

In the case where the rates *λ* and *μ* are constant over time, simple observables of the BD process such as survival probabilities or average population are analytically tractable and may be computed explicitly. For more intricate observables (in particular, topological properties of the resulting trees), or when the rates *λ* and *μ* are time-dependent, it becomes tedious to solve the model analytically. However, it may always be computer simulated as an ABM, which is what we do. In our ABM, agents are manuscripts, starting initially with *N* of them. A time-step corresponds to a typical duration for a manuscript to be copied. At every time-step *t*, each extant manuscript has probability $\lambda_{t}$ to be copied and probability $\mu_{t}$ to be lost. Keeping track of the filiations between manuscripts, trees (or *arbres réels* in Fourquet’s words (47)) are built for each original manuscript, representing the genealogy of the full manuscript tradition stemming from that original. These trees are then reduced by only keeping the genealogical relationships between surviving witnesses, giving rise to the ideal stemma that a contemporary philologist would produce.

We posit that some of the characteristics of real manuscript populations may be captured by the relatively simple stochastic process described in the previous paragraphs. To probe whether this is indeed the case, we study this process by exploring its parameter space, building heatmaps obtained by varying the parameters *λ* (copy rate) and *μ* (loss rate) through ranges corresponding to reasonable orders of magnitude; here between $10^{-2}$ and $10^{-1}$. The survival rates and probability distribution of extant witnesses can be computed exactly as a function of the parameters. Other properties were computed by simulating a number $N=10^{4}$ of artificial manuscript traditions (that is, a realization of the stochastic process described above with $10^{4}$ original living manuscripts at t=0) for each pair of values (λ,μ), and for each of these traditions the corresponding stemma is constructed. We can then compute the average value of a variable of interest over the *N* populations: this gives the (λ,μ)-point in the heatmap.

The empirically observable variables that we examine are: the median final population of surviving witnesses for traditions with at least one witness; the extant lifespan of traditions (ie the distance in years between the oldest and youngest surviving witnesses); the bifidity ratio of stemmata; all of which can be compared to observed data from historical traditions. Additionally, we examine the survival rate of traditions (ie trees); the survival rate of manuscripts (ie nodes); as well as the median distance between the lowest common ancestor of the surviving witnesses and the actual root of the original tree (ie the original manuscript); all of which are not directly observable in historical data.

### A dynamic model of text transmission

The continuous-time Markov process that we consider in this work is a multiagent generalization of the birth-and-death process. The state of the system at any time is described by the number $M_{t}$ of agents (representing manuscripts) generated up to time *t* as well as the states $\{s_{i}{\}}_{0\leq i\leq M_{t}}$ of these agents with $s_{i}\in \{living,dead\}$. For each living agent *i* at time *t*, two transitions can occur during instant [t,t+dt]:

- A birth event with rate *λ*, such that with probability λdt a new agent (originating from *i*) is added to the system with state $s_{M_{t}+1}=living$,
- A death event with rate *μ* such that the state of *i* switches to $s_{i}=dead$ with probability μdt.

Starting with $M_{t=0}=1$ with a single living agent, we split the time evolution of the process into two phases:

- An active phase for $0\leq t\leq T_{\mathrm{act}}$ where both *λ* and *μ* are finite and constant
- A decimation or pure-death phase for $T_{\mathrm{act}}\leq t\leq T_{\mathrm{act}}+T_{\mathrm{inact}}$ where λ=0.

Moreover, we keep track of the parent–offspring relationship, so that the state of the system can be alternatively described by a directed tree with nodes standing for agents, where an edge (i,j) is drawn when agent *j* was generated by a birth event triggered by *i* (ie *j* was copied from *i*). We call a single realization of this process a *manuscript tradition*.

In the case of the constant rate model, the probability distribution of the number of surviving manuscripts in a tradition, as well as the survival rates of manuscripts and traditions have rather simple analytic expressions. Denoting by $t_{a}=T_{\mathrm{act}}$ the duration of the active phase and ${\bar{n}}_{t}=e^{(\lambda -\mu )t}$ the average number of living manuscripts (for $t\leq t_{a}$), the probability that a given tradition has *n* surviving witnesses at $t_{a}$ is given by Ref. (48)


$$P_{n}(t_{a})=\left\{ \begin{array}{cc} \;\;\;\;\;\;\;\;\;\;\;\;\;\;\;\;\;\;\;\;\;\;\;\;\;\;\;\;\;\;\;\;\;\;\;\;\;\;\;\;\;\frac{\mu }{\lambda }\eta_{t_{a}} & \text{if }n=0\\ \left(1-\frac{\mu }{\lambda }\eta_{t_{a}}\right)(1-\eta_{t_{a}})\eta_{t_{a}}^{n-1} & \text{if }n\geq 1 \end{array}\right.,$$

where


$$\eta_{t}=\frac{\lambda ({\bar{n}}_{t}-1)}{\lambda {\bar{n}}_{t}-\mu }.$$

Denoting now by $t_{e}=T_{\mathrm{inact}}$ the duration of the decimation phase, the probability that the final population of living manuscript is *n* given that there were *k* extant manuscripts at time $t_{a}$ is


P(N(te+ta)=n∣N(ta)=k)=(kk−n)(1−e−μte)k−ne−μnte

so that the final probability for the number of surviving witnesses for n≥1 is


Pn(ta+ta)=∑k=n∞(kk−n)(1−e−μta)k−ne−μntaPk(ta)=(1−μληta)1−e−μtaηta(eμtaηta(1−ηta)(eμta−1))−n.

while for n=0 the extinction rate of traditions—or equivalently the survival rate of works $s_{\mathrm{Works}}=1-P_{0}(t_{a}+t_{a})$, is


$$P_{0}(t_{a}+t_{a})=\frac{\mu }{\lambda }\eta_{t_{a}}+\left(1-\frac{\mu }{\lambda }\eta_{t_{a}}\right)\left(\frac{1}{\eta_{t_{a}}}-1\right)\frac{\eta_{t_{a}}(1-e^{\mu t_{a}})}{1-\eta_{t_{a}}(1-e^{\mu t_{a}})}.$$

The number of surviving witnesses follows a geometric distribution for n≥1, from which one can compute the median number of witnesses of surviving traditions.

It can also be shown (48) that the average cumulative population of a given tradition, ie the total number of manuscripts produced during the active phase, writes


$${\bar{M}}_{t_{a}}=1+\frac{\lambda }{\lambda -\mu }\left(e^{(\lambda -\mu )t_{a}}-1\right),$$

while the average number of living manuscripts at $t_{a}+t_{a}$ is


$${\bar{N}}_{t_{a}+t_{a}}=e^{(\lambda -\mu )t_{a}-\mu t_{a}}$$

so that the survival rate of manuscript over all traditions writes


$$s_{\mathrm{Man}}=\frac{{\bar{N}}_{t_{a}+t_{e}}}{{\bar{M}}_{t_{a}}}.$$

**Figure 5:**
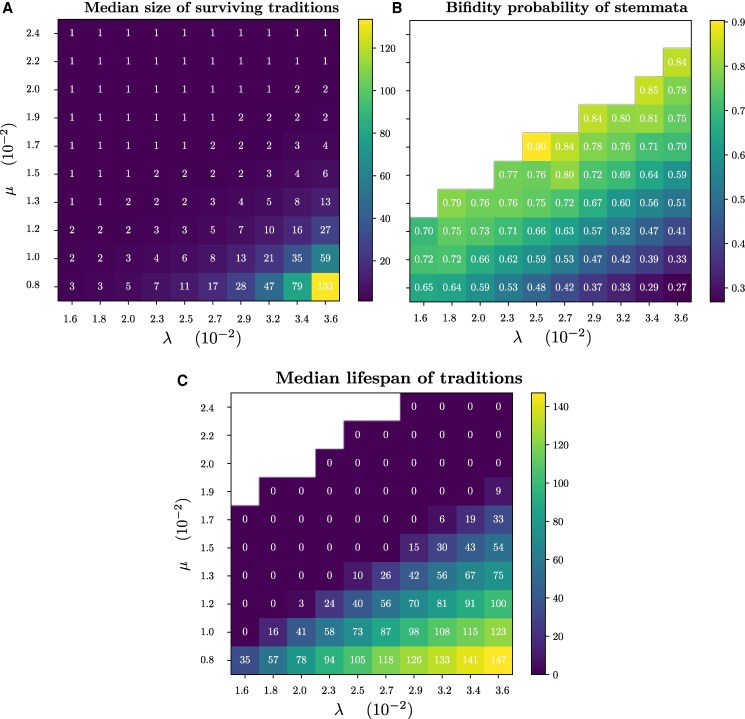
Heatmaps of model results for some empirically observable data, namely A) median number of surviving witnesses per tradition (ie surviving nodes per tree); B) proportion of stemmata with root out-degree equal to 2 (*bifid* stemmata); C) average lifespan of a tradition (difference in simulation years between oldest and newest witness birth time).

**Figure 6:**
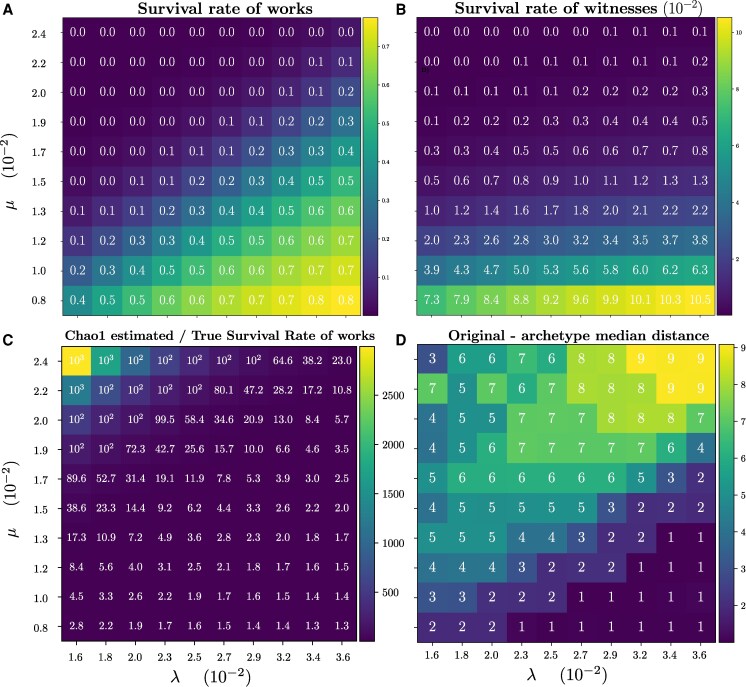
Heatmaps of model results for some nonempirically observable values, namely the survival rates computed from birth–death model for A) works (ie trees) and B) witnesses (ie nodes.); C) deviations between these survival rates computed exactly on the simulations, and those that would have been estimated using Chao1 on works; D) median distance in generations (number of nodes) between the archetype and the original, that is between the root of the full tradition’s tree and that of the stemma.

To explore the general behavior of the constant-rate model, across the space defined by the plausible ranges of the two parameters, we produce heatmaps for a number of relevant statistics, either observable or nonobservable in historical data (Figs. 5 and 6).

As for the other scenarii with variable rates or punctual decimation, they display a qualitatively similar behavior in parameter space, although the explicit expressions for the quantities computed above are more convoluted.

#### From simulated trees to stemmata

Once the full manuscript tradition originating from a given original is generated, one has to construct the corresponding stemma in order to allow for comparison with real philological data. This stemma should contain only surviving nodes of the full tree along with the minimal number of dead nodes needed to keep track of the genealogical relationship between those. In particular all terminal nodes (leaves) of the tree have to be surviving witnesses, and all internal (nonleaves) nodes need to have at least two direct children. Indeed, chains of nonbranching (parents of single child) hypothetical ancestors cannot be inferred from the comparative method and thus should not appear on a stemma by application of the parsimony principle.

**Figure 7:**
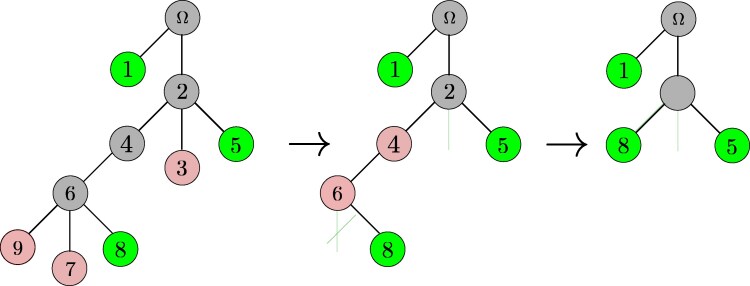
Steps of the algorithmic construction of the stemma from the full tree of a simulated tradition with original *Ω*, surviving witnesses in green and lost manuscripts in gray and red: removal of dead branches (left, nodes 3, 7, and 9), removal of nonbranching lost manuscripts (middle, nodes 4 and 6), final stemma (right).

The stemma is then constructed from the full tree by first recursively removing dead leaves until all branches have only surviving witnesses as terminal nodes, then removing chains of nonbranching dead nodes, until all remaining dead nodes have at least out-degree two (Fig. 7).

The resulting reduced tree then corresponds to the ideal (correct) stemma that would be reconstructed a posteriori by a philologist based on the systematic comparison of the witnesses of the tradition.

### Historically informed simulations

#### Total duration

We wish to model the transmission of medieval chivalric texts, that originated mostly in the years 1150–1350 and went out of fashion during the Renaissance, and then knew a period of inactive life (destruction, but almost no copy) until the beginning of preservation efforts during the Industrial Revolution. We therefore aim for a total duration of the simulation of 500 pseudoyears, split in two between an active period of 250 years and an equal inactive period of 250 years.

#### Order of magnitude for copy and loss rates in the simulations

Historical and philological knowledge of loss rates is very scarce and elusive. However, it is possible to approach them through different types of external evidence, such as data from ancient library catalogs (30), inventories or wills, as well as using intertextual evidence, such as allusions or references to unknown texts (49, 50) (Supplementary material). Most general estimates give a total loss of documents that is superior to 90%. Specifically for chivalric texts, some estimates give a total loss rate superior to 99% (Table S1). For our simulation needs, on this basis, we get a survival rate whose order of magnitude is between 0.1% and 10% in 500 years. From this, we can deduce a step loss rate for a given total survival rate. For instance, for 1% survival rate, $(1-\mu {)}^{T_{\mathrm{act}}+T_{\mathrm{inact}}}=0.01$, which simplifies to μ=0.0092. So, we retain values of *μ* between $10^{-2}$ to $10^{-1}$. Given that books could not have been produced order of magnitudes faster or slower than they were destroyed (or we would either drown in medieval manuscripts or have none), we explore the same range for *λ*, and assume a uniform prior distribution over these ranges in the inference procedure.

### Tree imbalance

Traditional philological studies have focused on the proportion of bifidity in stemmata collection. Yet, this measurement is relatively restricted in scope and does not reflect more general topological properties of the trees, and in particular their tendency to display imbalance at all levels. The characterization of imbalance is the object of a considerable literature in computational phylogenetics (29, 39), and a number of metrics have been proposed to quantify this property (51). In this work, we choose to focus on a single imbalance index for the stemmata, based on the subtrees generated by subsets of leaves representing witnesses of a tradition.

**Figure 8:**
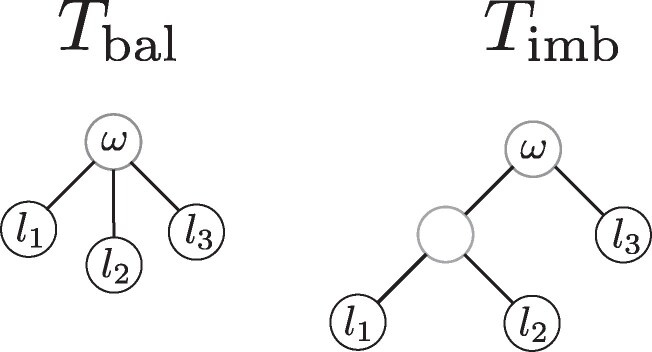
The two possible topologies of a three-leaves tree with only branching internal nodes.

There are only two possible topologies for a rooted tree with three leaves (forbidding internal nodes with a single child), one maximally imbalanced denoted by $T_{\mathrm{imb}}$, corresponding to the simplest “caterpillar tree” in the terminology of Fischer et al. (51), and one perfectly balanced $T_{\mathrm{bal}}$ with root node having three children (Fig. 8). Given a stemma with $N_{l}$ leaves, we construct the subtrees generated by all subsets of tree leaves $\left\{l_{1},l_{2},l_{3}\right\}$ which then fall into one of the two previously defined cases. Denoting by $n_{\mathrm{imb}}$ the number of generated subtrees having a $T_{\mathrm{imb}}$ structure, the imbalance index $i_{3}$ of the stemma is defined as the proportion of imbalanced subtrees


i3=nimb(Nl3).

This specific index, rather than other summary indices used to measure imbalance of tree topologies seems particularly fit to the study of stemmata as the construction of subtrees generated by witnesses is in itself an elementary emulation of the comparative method in philology, thus making the index readily interpretable. Besides, it allows for a rather scale-independent estimation of imbalance, as the root *ω* of a given subtree, or *hyparchetype* of the corresponding set of witnesses, ranges from low lying internal nodes to the actual archetype of the whole stemma when different subsets of witnesses are considered.

The value of this index on real stemmata (Table 2) is found to be equal to 0.93(±0.03), a value significantly higher than what is found in simulated stemmata for a wide range of parameters. Remarkably, the fact that empirical evolutionary data display a higher level of imbalance than cladograms generated by constant-rate birth-and-death models is also observed in biological phylogenetics (40).

### Observables of historical traditions

Data about the works and witnesses of medieval texts were collected from secondary literature and existing databases (32, 52–56) and reviewed using editions of the texts when they were available. Information on the stemmata is based on a restriction to the relevant genres of the data provided by the open-source collection OpenStemmata (57, 58). All used data (and relevant code) is provided as Supplementary information to this article.

#### Corpus and data collection

The corpus aims to encompass exhaustively all known longer chivalric narrative works in Old French (ie composed before c. 1340), namely *chansons de geste* (epics) and *romans* (romances) in verse and prose, as well as all the medieval manuscripts preserving them (be they in public institution or in private hands). These narratives are traditionally divided in three main thematic areas, or “matters”: matter of France (stories about Charlemagne, his lineage and his peers); matter of Britain (stories about Tristan or King Arthur and his knights); matter of “Rome” (stories inspired by Antiquity, and dealing with Alexander the Great, with the Fall of Troy and its consequences, or with Theban and Oriental myths). In addition from texts dealing with these matters, texts of the matter of England (stories of noble Anglo-Norman or English families) and of the Crusades were also included, as well as standalone adventures or courtly romances, containing similar narratives about knights and lovers. Shorter narrative forms, such as *lais* or *fabliaux*, were excluded, as well as romances not fitting in these thematic categories (*Roman de Renart*, and allegoric romances such as the *Roman de la Rose*).

The list of texts was established by crossing lists existing in the literature (52–54, 59), with existing databases (32) and online reference repositories (55, 56). When available, existing editions of individual texts were consulted, to verify the information and to collect existing stemmata. When no stemma was found in editions, scientific articles listed in the repositories were browsed to find them.

The notion of texts is relatively fluid in manuscript transmission, where every copy introduces innovations, that can range from simple copying errors to complete rewriting. Very often, a decision had to be made whether two versions of a given story constituted two versions of the same text or two distinct texts. To establish this, in ambiguous cases where information in repositories and reference works did not fully coincide, the following definition of text was used: a text is taken to be the formulation of a given story in human language, whose literary form and style can be detected and whose creation can be attributed to one or more individuals. Information for each text, witness and stemma was then recorded in the database (see Supplementary material for the complete set of rules and information).

### Extraction of summary statistics from historical data

In order to fit the model to real-world data, a subset of observables on manuscript traditions were selected for comparison with the model’s results:

- The total number of witnesses;
- The number of internal nodes of degree *k* for k=2,3,4 in the stemma of the tradition;
- The depth of the stemma, defined as the largest topological distance between the root and a leaf of the tree;
- The number of direct filiations between surviving witnesses in the stemma;
- The median lifespan of a tradition defined as the difference in age between the oldest and newest witness of a tradition. Since our model does not account for historical extrinsic factors on the variation of manuscript and work production rates, we expect this observable to be invariant enough by translation in time to allow for meaningful comparison with the model.

These summary statistics can be readily extracted from simulations, however some additional interpretative work is needed on empirical data.

Regarding temporal data, in order to convert collected evidence into distributions of creation date of manuscripts and lifespan of traditions, we assumed a uniform probability distribution for the birth time of a manuscript whenever its dating was given as an interval. Thus, for a manuscript with the creation date estimated to be within an interval $\left[y_{l},y_{u}\right]$, we assign the probability of creation $1/(y_{u}-y_{l})$ to each year within this range.

The difference in creation date of two witnesses dated in (potentially overlapping) ranges is then estimated by the expectation values of the difference between two random dates uniformly drawn from these ranges. Explicitly for two manuscripts dated in respective ranges [a,b] and [c,d], with a≤d, the difference *D* writes


$$D=\frac{1}{b-a}\frac{1}{d-c}\int_{a}^{b}\int_{c}^{d}|x-y|dxdy.$$

Summary statistics on stemmata was extracted from the DOT files available on OpenStemmata (58). Beforehand, stemmata were treated to be in an homogeneous format, by removing lateral transmission, as well as superfluous nodes conventionally represented on many published stemmata (ie nodes standing for hypothetical lost manuscripts, but having only an out-degree of one).

### Parameter estimation by simulation-based inference

Once the features just mentioned above have been extracted from empirical data, the parameters of the model are inferred using simulation-based inference, and more specifically the Neural Likelihood Estimation method (60) as implemented in the **sbi** python package (61). In this framework, a neural network model is first trained to emulate the simulator by predicting a vector of summary statistics x describing a single simulated tradition obtained from a set of parameters θ=(λ,μ). The summary statistics vector gathers the quantities described above. Once the model is trained on a large set of simulated data $\{\theta_{i},x_{i}{\}}_{i}$ for uniformly drawn parameters, the probability p(x|θ) that it predicts corresponding summary statistics given parameters values is used as an approximate of the likelihood function of the model. From there, one obtains the posterior probability distribution of parameters $p(\theta |X_{o})$ given a sample $X_{o}$ of summary statistics of empirical manuscript tradition, considered as a set of independent, identically distributed realizations of the birth–death process (assuming a uniform prior). The sample of empirical data used for inference is chosen randomly among works in our dataset so as to keep the same relative distribution of tradition sizes by witness number. Following this procedure, we are able to single-out relatively narrow regions of the parameter space for each scenario of the model.

As for the range of uniform prior distributions on parameter space, we use the intervals deemed reasonable based on historical data, namely $10^{-2}\leq \lambda ,\mu \leq 10^{-1}$.

As a posterior predictive check, we compute the features used for inference on new simulations realized with the median posterior value of the inferred parameters. These results are summarized in Table S5.

## Supplementary Material

pgag207_Supplementary_Data

## Data Availability

The data underlying this article are available at https://github.com/LostMa-ERC/ExtinctionOfTexts/, under a CC BY-SA 4.0 License, and archived on Zenodo, under doi:10.5281/zenodo.20555285.

## Notes

a There are also more pragmatic reasons for not including fragments in this research: very often, it is hard to assess if different fragments of a few leaves originally came or not from a unique now lost manuscript; moreover, due to their size, they usually offer very limited usable information in terms of date, origin, or even identity of the text they contain. Worse, due to their fragile nature, they still are often not identified or lost by curatorial institutions, limiting the quantity of information available to us today.

## Preprints

Preprints of this article are published at https://doi.org/10.48550/arXiv.2505.19246, and https://hal.science/hal-05086446v1/file/2505.19246v2.pdf.

## References

[1] Cisne JL . 2005. How science survived: medieval manuscripts’ “demography” and classic texts’ extinction. Science. 307:1305–1307. 15731453 10.1126/science.1104718

[2] Schlyter DCJ, Collins DHS. Corpus iuris sueo-gotorum antiqui. Samling af sweriges gamla lagar. Haeggström, 1827.

[3] Timpanaro S . La genesi del metodo del Lachmann, 4th ed. UTET Libreria, 2003.

[4] Einar Haugen O . 2 The genealogical method. In: Handbook of stemmatology. De Gruyter, 2020. p. 57–138.

[5] Segre C , editor. 1971. La chanson de roland. Documenti di filologia. Vol. 16. R. Ricciardi.

[6] Hoenen A . History of computer-assisted stemmatology. In: Handbook of stemmatology. De Gruyter, 2020. p. 294–303.

[7] Howe C, Windram H. Manuscript traditions. In: Kendal J, Kendal RL, editors. Oxford handbook of cultural evolution. Oxford University Press, 2025.

[8] Barbrook AC, Howe CJ, Blake N, Robinson P. 1998. The phylogeny of the *Canterbury Tales*. Nature. 394:839–839.

[9] Turnbull R . Codex Sinaiticus Arabicus and its family: a Bayesian approach. Brill, 2024.

[10] McCollum J, Turnbull R. 2024. Using Bayesian phylogenetics to infer manuscript transmission history. Digit Scholarsh Hum. 39:258–279.

[11] Hajič J Jr, Lanz V, Ballen GA. 2025. Genome of melody: applying bioinformatics to study the evolution of Gregorian chant. Philos Trans R Soc B: Biol Sci. 380:20240274. 10.1098/rstb.2024.0274 41537896

[12] Boyd R, Richerson PJ. Culture and the evolutionary process. University of Chicago Press, 1988.

[13] Fogarty L, Kandler A. Modelling cultural transmission. In: Kendal J, Kendal RL, editors. Oxford handbook of cultural evolution. Oxford University Press, 2025.

[14] Mace R, Holden CJ, Shennan S, editors. The evolution of cultural diversity: a phylogenetic approach. UCL Press/Left Coast Press, 2005.

[15] Graça da Silva S, Tehrani JJ. 2016. Comparative phylogenetic analyses uncover the ancient roots of Indo-European folktales. R Soc Open Sci. 3:150645. 26909191 10.1098/rsos.150645 PMC4736946

[16] Urban M . 2025. How oral traditions develop: a cautionary tale on cultural evolution from the Quechuan-speaking Andes. Humanit Soc Sci Commun. 12:1604. 41112410 10.1057/s41599-025-05335-4 PMC12534179

[17] Baumard N, Huillery E, Hyafil A, Safra L. 2022. The cultural evolution of love in literary history. Nat Hum Behav. 6:506–522. 35256800 10.1038/s41562-022-01292-z

[18] Morin O, Acerbi A. 2016. Birth of the cool: a two-centuries decline in emotional expression in anglophone fiction. Cogn Emot. 31:1–13. 10.1080/02699931.2016.1260528 27910735

[19] Alexander Bentley R, Carrington S, Ruck DJ. Modelling drift and selection in cultural evolution. In: Kendal J, Kendal RL, editors. Oxford handbook of cultural evolution. Oxford University Press, 2025.

[20] Bédier J . 1928. La tradition manuscrite du lai de l’ombre. Réflexions sur l’art d’éditer les anciens textes. Romania. 54:161–196 and 321–356.

[21] Alexander Baker C, Barbato M, Cavagna M, Greub Y, editors. L’ombre de Joseph Bédier: théorie et pratiques éditoriales au XXe siècle. ÉLiPhi, 2018.

[22] Duval F . La “Tradition manuscrite du Lai de l’Ombre” de Joseph Bédier ou la critique textuelle en question. Honoré Champion, 2021.

[23] Greg WW . 1931. Recent theories of textual criticism. Mod Philol. 28:401–404.

[24] Maas P . 1937. Leitfehler und stemmatische Typen. Byzantinische Z. 37:289–294.

[25] Castellani A . 1957.Bédier avait-il raison?: La méthode de Lachmann dans les éditions de textes du Moyen Âge. Leçon inaugurale donnée à l’université de Fribourg le 2 juin 1954. Discours universitaires, Nouvelle série. Vol. 20. Éditions Universitaires,

[26] Guidi V, Trovato P. 2004. Sugli stemmi bipartiti. Decimazione, asimmetria e calcolo delle probabilità. Filol Ital. 1:9–48.

[27] Hoenen A . Silva Portentosissima—Computer-Assisted Reflections on Bifurcativity in Stemmas. In: *Digital Humanities 2016: Conference Abstracts. Jagiellonian University & Pedagogical University* Kraków, DH 2016. Alliance of Digital Humanities Organizations, 2016. p. 557–560.

[28] Hoenen A, Eger S, Gehrke R. How many Stemmata with root degree *k*? In: *Proceedings of the 15th Meeting on the Mathematics of Language*. Association for Computational Linguistics, 2017. p. 11–21.

[29] Aldous DJ . 2001. Stochastic models and descriptive statistics for phylogenetic trees, from Yule to today. Statist Sci. 16:23–34.

[30] Buringh E . Medieval manuscript production in the Latin West. Brill, 2010.

[31] Kestemont M, Karsdorp F. Estimating the loss of medieval literature with an unseen species model from ecodiversity. In: *Proceedings of the Workshop on Computational Humanities Research*. CEUR. Vol. 2723. Gesellschaft fur Informatik, 2020. p. 44–55.

[32] Kestemont M, et al 2022. Forgotten books: the application of unseen species models to the survival of culture. Science. 375:765–769. 35175807 10.1126/science.abl7655

[33] Yessoufou K, Jonathan Davies T. Reconsidering the loss of evolutionary history: how does non-random extinction prune the tree-of-life. In: Pellens R, Grandcolas P, editors. Biodiversity conservation and phylogenetic systematics: preserving our evolutionary heritage in an extinction crisis. Topics in biodiversity and conservation. Springer International Publishing, 2016. p. 57–80.

[34] Chao A . 1984. Nonparametric estimation of the number of classes in a population. Scand J Stat. 11:265–270.

[35] Weitzman MP . 1987. The evolution of manuscript traditions. J R Stat Soc Ser A. 150:287–308.

[36] Yule GU . 1925. A mathematical theory of evolution, based on the conclusions of Dr. J. C. Willis. Phil Trans R Soc Lond B. 213:402–410.

[37] Camps J-B, et al Make love or war? Monitoring the thematic evolution of medieval French narratives. In: Šeļa A, Jannidis F, Romanowska I, editors. *Proceedings of the Computational Humanities Research Conference 2023 Paris*; France, December 6–8, 2023, CEUR Workshop Proceedings, Paris. Vol. 3558. Gesellschaft fur Informatik, 2023. p. 734–756.

[38] Delsaux O . Manuscrits et pratiques autographes chez les écrivains français de la fin du Moyen Âge: L’exemple de Christine de Pizan. Librairie Droz, 2013.

[39] Mooers AO, Heard SB. 1997. Inferring evolutionary process from phylogenetic tree shape. Q Rev Biol. 72:31–54.

[40] Khurana MP, Scheidwasser-Clow N, Penn MJ, Bhatt S, Duchêne DA. 2024. The limits of the constant-rate birth-death prior for phylogenetic tree topology inference. Syst Biol. 73:235–246. 38153910 10.1093/sysbio/syad075 PMC11129600

[41] Blythe RA, McKane AJ. 2007. Stochastic models of evolution in genetics, ecology and linguistics. J Stat Mech. 2007:P07018.

[42] Castellano C, Fortunato S, Loreto V. 2009. Statistical physics of social dynamics. Rev Mod Phys. 81:591–646.

[43] Kandler A, Powell A. 2018. Generative inference for cultural evolution. Philos Trans R Soc B: Biol Sci. 373:20170056. 10.1098/rstb.2017.0056 PMC5812969 29440522

[44] Weitzman MP . 1982. Computer simulation of the development of manuscript traditions. ALLC Bull. 10:55–59.

[45] Cranmer K, Brehmer J, Louppe G. 2020. The frontier of simulation-based inference. Proc Natl Acad Sci USA. 117:30055–30062. 32471948 10.1073/pnas.1912789117 PMC7720103

[46] Norris JR . Markov chains. Cambridge University Press, 1998.

[47] Fourquet J . 1945. Le paradoxe de Bédier. Mélanges: Études Littéraires. II:1–16.

[48] Kendall DG . 1948. On the generalized “birth-and-death” process. Ann Math Statist. 19:1–15.

[49] Wilson RM . The lost literature of medieval England. Methuen, 1952.

[50] Bardon H . La littérature latine inconnue. C. Klincksieck, 1952.

[51] Fischer M, Herbst L, Kersting S, Luise Kühn A, Wicke K. Tree balance indices: a comprehensive survey. Springer, 2023.

[52] Camps J-B . 2016. *La “Chanson d’Otinel”: édition complète du corpus manuscrit et prolégomènes à l’édition critique*. hèse de doctorat, dir. Dominique Boutet, Paris-Sorbonne, Paris.

[53] Andrea Martina P . 2018. *La produzione manoscritta del romanzo francese in versi: modelli materiali e modelli di cultura*. Thèse de doctorat, Sorbonne Université.

[54] Vitale-Brovarone A . La diffusion manuscrite des chansons de geste: une vue d’ensemble. In: Helkkula M, Välikangas O, editors. Tra Italia e Francia. Entre France et Italie. In honorem Elina Suomela-Härmä. Mémoire de la Société Néophilologique de Helsinki. Vol. 69. Société Néophilologique, 2006. p. 473–488.

[55] Leurquin Anne-Françoise, Savoye M-L. *Jonas: Répertoire des textes et des manuscrits médiévaux d’oc et d’oïl*. Institut de recherche et d’histoire des textes-CNRS, Paris and Orléans; 1993-2026. http://jonas.irht.cnrs.fr/.

[56] Brun L , editor. Arlima - Archives de littérature du Moyen Âge. Université d’Ottawa, 2005.

[57] Camps J-B, Gabay S, Fernández Riva G. Open stemmata: a digital collection of textual genealogies. In: *EADH2021: Interdisciplinary Perspectives on Data, 2nd International Conference of the European Association for Digital Humanities*. Krasnoyarsk; 2021.

[58] Camps J-B, Fernandez Riva G, Gabay S. Open stemmata: database, November 2021. https://github.com/OpenStemmata/database/.

[59] Hasenohr Geneviève, Zink M, Bossuat R, Pichard L, Raynaud de Lage G, editors. Dictionnaire des lettres françaises: le Moyen Âge. Fayard, 1994.

[60] Papamakarios G, Sterratt D, Murray I Sequential neural likelihood: fast likelihood-free inference with autoregressive flows. In: Kamalika Chaudhuri, Masashi Sugiyama, editors, *Proceedings of the Twenty-Second International Conference on Artificial Intelligence and Statistics*, *Proceedings of Machine Learning Research*. Vol. 89. PMLR, 2019. p. 837–848.

[61] Tejero-Cantero A, et al 2020. SBI: a toolkit for simulation-based inference. J Open Source Softw. 5:2505.

