# Supplementary material for: On the transmission of texts: Written cultures as complex systems
Source: PNAS Nexus. 2026 Jul 7;5(7):pgag207. doi: 10.1093/pnasnexus/pgag207 (PMC13339094; doi:10.1093/pnasnexus/pgag207)
Supplement: pgag207_Supplementary_Data [file pgag207_supplementary_data.pdf]

Supplementary Material to
On the Transmission of Texts: Written Cultures as Complex Systems

Jean-Baptiste Camps, Julien Randon-Furling, Ulysse Godreau

Supplementary Methods

Loss rates of manuscripts and incunabula

Copy (or birth) and loss (or death) rates

Gathering historical information on loss rates is a difficult issue. We give here some estimates that have been provided on the basis of ancient catalogues, known print runs of *incunabula*, as well as other diverse methods.

Ancient catalogues. Using data based on a large collection of historical catalogues of manuscripts, Buringh [5] provides estimates for the Latin West, with a geometric mean of loss around -25% per century, with variations from -11% in the 9th to -32% in the 14th and 15th centuries (with local variations between medieval institutions from –3% to –71% per century). The global loss rate for non-illustrated manuscripts of several well known collections has been estimated around 93-97% [13, 16, 20, 17]. But estimations based on well known institutional collections, from which some manuscripts are known to have survived, are potentially biased. Trying to account for fully lost libraries, Buringh [5] is compelled to revise his estimates higher, to -25% by century until the 12th, up to -43% in the 15th.

Moreover, generally speaking, using catalogues leads to several biases that can lead to an underestimation of the loss rates:

1. medieval catalogues are produced mostly by institutions, and among them mostly ecclesiastical institutions;
2. they concern mostly Latin religious texts, that are presumed to have been less decimated than vernacular leisure literature;
3. catalogues reveal the existence of a preservation effort;
4. catalogues do not necessarily record all books, only the ones that are deemed most worthy of description and preservation.

All this can lead to underestimate the loss rate, and is also tributary to the fate of ecclesiastical institutions. In France, where many of them were maintained until the Revolution, and their assets then seized and included in national collections, the loss rate estimated from Buringh's data collection [5] is 72%. In England, where the Dissolution caused the scattering and destruction of many ecclesiastical collections, the loss rate is 91%. In addition, the few data that we have concerning non ecclesiastical collections, that included more vernacular literature, seem to confirm this bias: the loss rate for the Royal Library of the Louvres, in France, is of 92% [5], while, for the few examples of aristocratic libraries recorded by Buringh, it reaches 100% loss.

In addition to the institutional nature of the library preserving them or the nature of the text, the monetary value of books also causes important variations in survival rates [4], in particular when they are richly decorated with miniatures and other paintings. For instance, concerning the luxury collection of the dukes of Burgundy, Wijsman [20] notes both a low destruction rate (41% for the books in the 1487 catalogue), but also notes that those catalogues tended to exclude books of lower values.

Print runs of early prints. For incunabula, using editions whose original number of copies made is known, it is possible to gather loss estimates by counting known surviving exemplars in public or private collections: doing so for Venetian incunabula, Trovato [19] finds very variable loss rates according to textual and material typology, from 73% for the *Decretales* printed on parchment to 99.3% for more popular chivalrous literature (*Orlando furioso* for instance). This shows the importance both of variation in time and space, and of textual contents and material typology. A more general estimate for 15th century prints yields a loss rate of 95.7% for Europe [15].

Other estimates. In some extreme cases, loss can be very close to 100%, for reasons that may combine the fragility of the document form, lack of consideration for the documents or large scale historical events such as political instability, invasions or major cultural changes; examples are provided by cases as different as the Merovingian royal diplomas on papyrus or the Lombard royal charters [10], the Mayan (Pre-Columbian) manuscripts or medieval notarial acts [12]. Production estimates have also been attempted on the basis of the quantity of sealing wax acquired by a given producer (a chancellery for instance [1]). More founded loss estimates have also been gathered by counting how many of the acts mentioned in imperial or royal registers are kept in original or consigned in the archives of the recipients: this gives a loss rate of originals varying from 80% (acts from the emperor Charles IV in 1360-1361) to 90% for the acts from Louis X of France, increasing to 99% for the judgments rendered by his Parliament, suggesting here as well a massive effect of typological variation [12, 8], resulting in very strong biases in the body of documents available to us.

The loss rates indicated in Table S1 and Table S2 are drawn from the bibliography. Their original sources include, for manuscripts (*MSS*), medieval catalogues (mostly of ecclesiastical institutions), where the number of original entries is compared to the number of (identifiable) surviving manuscripts; for early printed books (*Pr*), the print run of early *incunabula editions*; as well as, for reference, the results of different mathematical models. The sample size (N) is equal to the estimated total original population, before decimation.

Table S1 presents the most relevant data to our case study (medieval vernacular chivalric literature), while Table S2 gives other estimates for comparative purposes.

In Table S2, the final estimates of manuscript loss for the Holy Roman Empire of the German Nation and for Europe are based on the observed loss of contemporary *incunabula*, while trying to accommodate for historical factors specific of manuscript production and conservation.

Bifidity ratio in historical stemmata collections

Table S3 gives the computed bifidity ratio in stemmata collections since Bédier. When available, are additionally given the number of trees analysed (N), the restrictions in terms of language(s), literary genre, status of the trees (final tree, used as basis of text editions versus provisional tree) and the criteria used for inclusion in the collection, as well as the reference to the source.

Data collection

Two versions of the same story were taken to be different texts when:

- they differ in terms of language (e.g., Continental French vs. Anglo-French);
- they differ in terms of form: prose vs. verse; octosyllabic versus decasyllabic verses; assonance vs. full rhyme;
- they differ in authorship (a different individual is associated with their creation);
- they substantially differ in terms of story, or their texts cannot be aligned (i.e. there are no significantly matching text portions).

For each text, the following information was collected, in addition to author and title:

- date of creation (usually estimated based on historical, linguistic and literary evidence);
- witnesses;
- stemma.

The stemmata were encoded in DOT format, and submitted to an open repository using their set of conventions for representing surviving and lost nodes, as well as direct or lateral transmission and uncertainty [7]. In addition, the following information was collected about the witnesses:

- date of creation (usually estimated based on material, linguistic and paleographic evidence, sometimes known through declarations by the scribe, known as “colophons”);
- curatorial information: institution of preservation and shelfmark;
- status (complete, mutilated or fragmentary).

Most of the time, the dates of the texts or of the witnesses are not known precisely, but estimated. Estimates made by experts are usually expressed in relatively vague form, in prose. We opted to convert them to explicit ranges, following this set of rules:

Exact dates exact dates, sometimes given by the scribe of the manuscript in the colophon, are followed;
Explicit Range the range given is kept;
Non explicit range a range of 20 years is used, bounded or centered according to the information given;
Cumulative ranges both ranges are included. When the ranges given are not continuous, the in-between period is added;
Fuzzy date/range a period of 10 years (5 before, 5 after) is added to the date or range expressed ;
Single delimiter pseudo-range i.e., terminus ad quem/post quem without explicit range) are treated similarly as fuzzy dates;
Exotic modifiers such as “at the latest” are ignored for now;
Competing dating when two dating are given, the most precise is used.

Example of conversions are given in Table S4.

Supplementary Table S1. Estimates of loss rates of documents from various sources for chivalric vernacular texts

Ref	Type	Source	Date	Region	Lang.	Loss (%)	N
[6]	MSS	catalogues	1100-1500	UK	Fre.	82.00	45
[19]	Pr	print run	1495-1532	Italy	Ita.	99.31	5200
[14]	MSS	*unseen sp.*	800-1500	West. Eur.	vern.	≥91.00	41 244
[14]	MSS	*unseen sp.*	1100-1500	West. Eur.	Fre.	≥94.60	27 278

Supplementary Table S2. Estimates of loss rates from various sources (all other types) – starred languages means they constitute the majority of the collection, but that some other languages may be marginally present

Ref	Type	Source	Date	Region	Lang.	Loss (%)	N
[5]	MSS	catalogues	700-1500	UK	Lat.*	90.83	17207
[5]	MSS	catalogues	700-1500	France	Lat.*	72.01	11645
[19]	Pr	print run	1476-1542	Italy	Ita.	92.39	14700
[19]	Pr	print run	1495-1532	Italy	Lat.	94.56	3300
[15]	Pr	print run	1453-1500	Europe	all	95.70	18488000
[15]	Pr	print run	1453-1500	Centr. Eur.	all	94.50	6144000
[15]	MSS	*estimate*	1400-1500	Empire	all	≥95	1122000
[15]	MSS	*estimate*	1400-1500	Europe	all	≥95	≥ 2396000

Supplementary Table S3. Bifidity estimates in different stemmata collections, since Bédier

bifid (%)	N	Lang.	Genre restr.	Status	Collection	Ref.
95.5	110	Fr, Lat, Eng, Ger	no	NA	NA	[2]
69.0	130	Pro	troubadour lyric	NA	NA	[18]
75.5	94	Fr	no	all	works cited in [3]	[9]
82.5	86	Fr	no	final	works cited in [3]	[9]
83.1	89	Non	no	final	*Bibl. et Ed. Arnamagn.*	[11]
77.0	117	Fr	epics, romances	all	*OpenStemmata*	[7]

Supplementary Table S4. Example of conversion of prose dating to ranges

Prose	Range
Exact dates	
17th may 1423	1423
Explicit range	
12th century	1101-1200
2nd half of the 12th century	1151-1200
14th century, after 1339	1340-1400
Non explicit range	
end of the 12th century	1181-1200
middle of the 12th century	1141-1160
first decades of the 14th century	1301-1320
Cumulative ranges	
end of the 12th or beg. of the 13th c.	1181-1220
beg. or 3rd quarter of the 15th c.	1401-1475
Fuzzy date or range	
circa 1240	1236-1245
around the end of the 12th c.	1176-1205
Single delimiter pseudo-range	
after 1460	1461-1470
before 1460	1451-1460
Exotic modifiers (ignored)	
beg. of the 13th c. at the latest	1201-1220
Competing dating	
2nd quarter of the 13th c., c. 1240	1236-1245

Supplementary Table S5. Posterior checks of the three models. Observed features are computed on simulated traditions, taking the median of the posterior distribution as running parameters. For each of the features identified in section *Parameter estimation by simulation-based inference*, the median/interquartile range or mean/standard deviation of the observable is computed for 10^5 simulated traditions with infered parameters. These features are the median number of witnesses, of nodes (witnesses and hypothetical intermediaries), the lifespan (time separating the oldest and most recent witnesses), as well as the number of nodes with outdegrees 1 (direct witnesses connections), 2 (bifidity), 3 and 4, and finally the depth (longest path between the root and any leaf).

**feature**	**empirical**		**constant-rate**		**decay**		**decimation**	
	median	*IQR*	*median*	*IQR*	*median*	*IQR*	*median*	*IQR*
wit. nb.	2	8	**3**	4	**3**	4	4	5
nodes nb.	16	12	8	7	8	7	**9**	8
lifespan	61	123	49	89	70	118	**60**	95
	mean	*std*	*mean*	*std*	*mean*	*std*	*mean*	*std*
$n_{d=1}$	0.24	0.68	0.48	0.72	**0.36**	0.61	0.85	1.03
$n_{d=2}$	5.96	3.53	3.04	2.15	2.77	1.95	**3.12**	2.33
$n_{d=3}$	0.8	1.2	0.64	0.82	0.62	0.79	**0.68**	0.89
$n_{d=4}$	0.2	0.43	**0.13**	0.36	**0.13**	0.36	**0.13**	0.37
depth	5.4	1.7	4.1	1.3	3.9	1.7	**4.2**	1.3

References

1. Robert-Henri Bautier. Introduction. In André Lapeyre and Rémy Scheurer, editors, *Les notaires et secrétaires du roi sous les règnes de Louis XI, Charles VIII et Louis XII, 1461-1515*, volume 1, pages IX–XXXIX. Bibliothèque nationale, Paris, 1978.
2. Joseph Bédier. La tradition manuscrite du lai de l'ombre. Réflexions sur l'art d'éditer les anciens textes. *Romania*, 54:161–196 and 321–356, 1928.
3. Robert Bossuat. *Manuel bibliographique de la littérature française du Moyen Âge*. Bibliothèque elzévirienne, Études et documents. Librairie d'Argences, Paris, 1951.
4. Carla Bozzolo and Ezio Ornato. *Pour une histoire du livre manuscrit au Moyen Âge: trois essais de codicologie quantitative*. Éditions du Centre national de la recherche scientifique Paris, France, 1980.
5. Eltjo Buringh. *Medieval Manuscript Production in the Latin West*. Brill, Leiden, 2010.
6. Jean-Baptiste Camps. *La 'Chanson d'Otinel': édition complète du corpus manuscrit et prolégomènes à l'édition critique*. thèse de doctorat, dir. Dominique Boutet, Paris-Sorbonne, Paris, 2016.
7. Jean-Baptiste Camps, Gustavo Fernandez Riva, and Simon Gabay. Open Stemmata: Database, November 2021. `https://github.com/OpenStemmata/database/`.
8. Olivier Canteaut. Quantifier l'activité des chancelleries à l'aune de la tradition des actes : l'exemple de la chancellerie des derniers Capétiens (1314-1328). In Olivier Canteaut, Olivier Guyotjeannin, and Olivier Poncet, editors, *Actes royaux et princiers à l'ère du numérique (Moyen Âge-Temps modernes)*, pages 103–114, Pau, 2020.
9. Arrigo Castellani. *Bédier avait-il raison?: La méthode de Lachmann dans les éditions de textes du Moyen Âge. Leçon inaugurale donnée à l'université de Fribourg le 2 juin 1954*. Number 20 in Discours universitaires, Nouvelle série. Éditions Universitaires, Fribourg, 1957.
10. David Ganz and Walter Goffart. Charters Earlier than 800 from French Collections. *Speculum*, 65(4):906–932, 1990.
11. Odd Einar Haugen. The silva portentosa of stemmatology: Bifurcation in the recension of Old Norse manuscripts. *Digital Scholarship in the Humanities*, 31(3):594–610, 2015.
12. Eberhard Holtz. Überlieferungs- und Verlustquoten spätmittelalterlicher Herrscherurkunden. In Mathias Lawo and Olaf B. Rader, editors, *Turbata per aequora mundi: Dankesgabe an Eckhard Müller-Mertens*, pages 67–80. Harrassowitz, Hanover, 2001.
13. Mike Kestemont and Folgert Karsdorp. Estimating the Loss of Medieval Literature with an Unseen Species Model from Ecodiversity. In *Proceedings of the Workshop on Computational Humanities Research*, volume 2723 of *CEUR*, pages 44–55, 2020.
14. Mike Kestemont, Folgert Karsdorp, Elisabeth de Bruijn, Matthew Driscoll, Katarzyna A. Kapitan, Pádraig Ó Macháin, Daniel Sawyer, Remco Sleiderink, and Anne Chao. Forgotten books: The application of unseen species models to the survival of culture. *Science*, 375(6582):765–769, February 2022.
15. Uwe Neddermeyer. Möglichkeiten und Grenzen einer quantitativen Bestimmung der Buchproduktion im Spätmittelalter. *Gazette du livre médiéval*, 28(1):23–32, 1996.
16. Uwe Neddermeyer. *Von der Handschrift zum gedruckten Buch: Schriftlichkeit und Leseinteresse im Mittelalter und in der frühen Neuzeit quantitative und qualitative Aspekte*. phdthesis, Philosophische Fakultät Köln, Wiesbaden, 1998.
17. Frits van Oostrom. *Stemmen op schrift: geschiedenis van de Nederlandse literatuur vanaf het begin tot 1300*. Bert Bakker, Amsterdam, 2013.
18. William P. Shepard. Recent theories of textual criticism. *Modern Philology*, 28(2):129–141, 1930.
19. Paolo Trovato. *Everything you always wanted to know about Lachmann's method: a non-standard handbook of genealogical textual criticism in the age of post-structuralism, cladistics, and copy-text*. Libreriauniversitaria.it edizioni, Limena, 2014.
20. Hanno Wijsman. *Luxury Bound: Illustrated Manuscript Production and Noble and Princely Book Ownership in the Burgundian Netherlands (1400-1550)*, volume 16 of *Burgundica*. Brepols Publishers, Turnhout, 2010.
